# Astragalus Polysaccharides Lowers Plasma Cholesterol through Mechanisms Distinct from Statins

**DOI:** 10.1371/journal.pone.0027437

**Published:** 2011-11-16

**Authors:** Yunjiu Cheng, Kai Tang, Suhua Wu, Lijuan Liu, Cancan Qiang, Xiaoxiong Lin, Bingqing Liu

**Affiliations:** Division of Cardiology, the First Affiliated Hospital, Sun Yat-Sen University, Guangzhou, China; Heart Center Munich, Germany

## Abstract

To determine the efficacy and underlying mechanism of Astragalus polysaccharides (APS) on plasma lipids in hypercholesterolemia hamsters. The effect of APS (0.25g/kg/d) on plasma and liver lipids, fecal bile acids and neutral sterol, cholesterol absorption and synthesis, HMG-CoA reductase activity, and gene and protein expressions in the liver and small intestine was investigated in twenty-four hypercholesterolemia hamsters. Treatment periods lasted for three months. APS significantly lowered plasma total cholesterol by 45.8%, triglycerides by 30%, and low-density lipoprotein-cholesterol by 47.4%, comparable to simvastatin. Further examinations revealed that APS reduced total cholesterol and triglycerides in the liver, increased fecal bile acid and neutral sterol excretion, inhibited cholesterol absorption, and by contrast, increased hepatic cholesterol synthesis and HMG-CoA reductase activity. Plasma total cholesterol or low-density lipoprotein-cholesterol levels were significantly correlated with cholesterol absorption rates. APS up-regulated cholesterol-7α-hydroxylase and LDL-receptor gene expressions. These new findings identify APS as a potential natural cholesterol lowering agent, working through mechanisms distinct from statins.

## Introduction

Hypercholesterolemia is a contributing factor to atherosclerosis and consequent cardiovascular and cerebrovascular disease. Clinically, statins effectively lower plasma cholesterol by inhibiting HMG-CoA reductase activity [1]. Nevertheless, some patients under statin treatment can not tolerate statins well or do not reach the low-density lipoprotein-cholesterol (LDL-C) goal recommended by the US National Institutes of Health guidelines [2]. Therefore, it is desirable to develop natural drugs that have cholesterol-lowering effect comparable to statins, but could be tolerated well by the patients.

Astragalus polysaccharides (APS), an extract of Radix Astragali, is one of the main efficacious principles. APS-I and APS-II are well known to be the major structural components of APS. APS-I (molecular weight = 1,699,100 Da) consists of arabinose and glucose in a molar ratio of 1∶3.45, while APS-II (molecular weight = 1,197,600 Da) consists of rhamnose, arabinose and glucose in a molar ratio of 1∶6.25∶17.86 [3]. In China, APS has been extensively used to treat viral infection [4], [5], acute myocarditis [6], glomerulonephritis [7], diabetes [8], tumor [9], and many other illnesses, with no toxic record in clinic.

Previous reports indicated that some dietary soluble polysaccharides lower plasma cholesterol via reduction of intestinal cholesterol absorption [10], [11], [12] or interference with the enterohepatic circulation of bile acids [13], [14]. However, the cholesterol-lowering effect of APS has not been well studied and underlying mechanisms behind are still elusive. Hence, we are here to determine how APS regulates plasma cholesterol and the cholesterol metabolic pathways in hyperlipidemia hamsters.

## Materials and Methods

### Animals and diets

Twenty-four male Golden Syrian hamsters weighing 130–140 g (Animal experimental center, Guangdong, China) were housed one per cage with a 12 h light: dark cycle and humidity 50–60%. The hamsters were fed a regular rodent cholesterol-free chow, with free access to food and water. After two weeks of adaptation, animals were weighed and randomly divided into 3 groups (n = 8/group): APS group, Simvastatin group and Control group. The food of all the hamsters was switched to a high-fat diet for a period of 3 months. The diet contained 10% lard, 10% yoke powder, 1% cholesterol and 79% standard chow mix (Animal experimental center, Guangdong, China). Diets were prepared once for the first two weeks and on a weekly basis thereafter. For the next 3 months, based on the high-fat diet, animals in APS group and Simvastatin group were given orally APS at 0.25 g/kg/d and simvastatin at 10 mg/kg/d, respectively. Control animals received an equal volume of vehicle (0.9% saline). The purity of APS was >95% (Dabang animal Pharmaceutical Co, Ltd. Inner Mongolia, China). Simvastatin tablets were obtained from Merck & Co., Inc. USA. The study protocol was approved by the ethics committee of the First Affiliated Hospital of Sun Yat-Sen University (Guangzhou, China).

Blood (0.5 mL) was collected from the tail vein into a heparinized capillary tube at the end of month 0,1.5, 3, 4.5, and 6 after overnight fasting. Plasma was harvested for plasma lipids and plasma liver enzyme measurements. At the end of month 0, 3, 6, fecal samples collected over 4 days for each animal were freeze- dried, weighed, and ground to powder for bile acid and neutral sterol analysis. A week prior to sacrifice, the hamsters were gavaged with 2 µCi [^3^H]-β-sitosterol and 1 µCi [^14^C]-cholesterol, and the feces were collected for three days for fractional cholesterol absorption measurement. One hour before sacrifice, 40 mCi [^3^H] water was injected intraperitoneally for hepatic cholesterol synthesis rate measurement. At the end of the experiment, the hamsters were anesthetized with an i.p. injection of sodium pentobarbital (40 mg/kg; Shanghai Chemical Reagent Company, Beijing, China) and their livers and intestines obtained were immediately frozen and stored at −80°C for histological examination and measurement of lipids, HMG-CoA reductase activity as well as gene and protein expressions.

### Measurements of plasma and liver lipids and plasma liver enzyme

Plasma concentrations of total cholesterol (TC), triglycerides (TG), high-density lipoprotein-cholesterol (HDL-C) and low-density lipoprotein-cholesterol (LDL-C) were measured in triplicate using kit methods enzymatically following the instructions provided (Xueyou Biotechnology Company, Guangzhou, China). Total lipids were extracted from 0.5 g liver tissue in chloroform/ methanol (vol/vol  = 2/1). Liver TC and TG were measured using the same method and reagents for plasma cholesterol [15]. Plasma concentration of liver enzyme, alanine aminotransferase (ALT) and aspartate aminotransferase (AST) were analyzed with kit method (Xueyou Biotechnology Company, Guangzhou, China).

### Fecal bile acid and neutral sterol excretion

The total bile acid content in 0.5 g of the ground feces was determined using a 3α-hydroxysteroid dehydrogenase assay [16]. A second 0.5 g aliquot of feces were saponified with alkaline and ethanol. Samples were then extracted in 15 ml of petroleum ether to which 1.0 mg of 5-cholestene (Sigma) had been added as an internal standard. Mass analysis of the extracted neutral sterols was conducted by gas-liquid chromatography as described previously [17].

### Fractional cholesterol absorption

Fractional cholesterol absorption was measured by the fecal isotope ratio method described by Turley et al [18]. At the end of the experiment, the hamsters were gavaged with 2 µCi of [^3^H]-β-sitosterol (American Radiolabeled Chemicals, St. Louis, MO) and 1 µCi of [^14^C]-cholesterol (Amersham Pharmacia, Piscataway, NJ) dissolved in 100 µl of soybean oil. Each animal was individually housed in a cage with a wire bottom and was allowed free access to diet and water for 3 days. The feces were collected for three days, 1 g of feces from each animal was was extracted with chloroform/ methanol (vol/vol  = 2/1). The extracts were transferred to a scintillation vial and dried at 65°C under N_2_. The [^14^C] cholesterol and [^3^H] sitosterol counts were measured in a liquid scintillation counter(Beckman, Palo Alto, CA). Fractional cholesterol absorption was calculated using the following equation: (^14^C/^3^H [dose]–^14^C/^3^H [fecal sample])/(^14^C/^3^H [dose])×100.

### Hepatic cholesterol synthesis rate

The rate of in vivo cholesterol synthesis in liver was measured as described [16]. At the end of the experiment, animals were given an i.p. of 40 mCi of [^3^H] water (New England Nuclear Corp.), and anesthetized, exsanguinated after 1 hour. Aliquots of liver were saponified, and the radiolabeled digitonin-precipitable sterols were extracted and measured. Cholesterol synthesis rates were expressed as the nanomoles of [^3^H] water incorporated in digitonin-precipitable sterols per hour per gram wet weight of tissue.

### Liver HMG-CoA reductase activity measurement

Frozen liver (400 mg) was homogenized and liver microsomal protein was isolated by differential centrifugation, resuspended, divided into several aliquots as previously described [19]. Liver HMG-CoA reductase activity was determined according to the method described by by van Heusden and Wirtz [20].

### Measurement of gene expressions in the liver and small intestine

Total RNA was extracted from liver (left lateral lobe) or small intestine (jejunum) using TRIzol reagent (Invitrogen, USA). An aliquot (1 µg) of RNA was transcribed to cDNA using Ominiscript reverse transcriptase (Roche, USA) under the following conditions:. 37°C for 1 h and 93°C for 5 min. The cDNA was diluted 1∶10 using diethyl pyrocarbonate water, and the real-time PCR reaction solution consisted of 5 µl cDNA, 12.5 µl QuantiTect SYBR Green PCR kit (Roche, USA), 5.5 µl diethyl pyrocarbonate water, and 1 µl forward and reverse primer (20 pmol) for a final reaction volume of 25 µl. The primer sequences are presented in Table 1. Quantitative real-time PCR analysis was performed on an Applied Biosystems 7500 real-time PCR machine (Applied Biosystems, USA) using the following conditions: 50°C for 2 min, 94°C for 10 min, and 40 cycles of 94°C for 10 s and 60°C for 1 min. The fluorescence measurement used to calculate threshold cycle (Ct) was made at the 60°C point. A dissociation curve was run at the end of the reaction to ensure a single amplification product. One sample from the control was used as an external calibrator. Gene expression was normalized to cyclophilin mRNA concentration and presented as fold of the control by setting the expression of the control group to 1.00.

**Table 1:** Primer sequences used for real-time PCR analysis.

Gene	Primer sequence	NCBI Accession Number
cyp7α-1	5′-ACCTGCCGGTACTAGACAGCA-3′	NM_ 012942
	5′-TGCGGATATTCAAGGATGCA-3′	
LDL-R	5′-ACCTGCCGGTACTAGACAGCA-3′	NM_175762
	5′-GAACTTGGGTGAGTGGGCAC-3′	
NPC1L1	5′-CCTGACCTTTATAGAACTCACCACAGA-3′	DQ897680
	5′-GGGCCAAAATGCTCGTCAT-3′	
ABCG5	5′-TGATTGGCAGCTATAATTTTGGG-3′	AF312713
	5′-GTTGGGCTGCGATGGAAA-3′	
ABCG8	5′-TGCTGGCCATCATAGGGAG-3′	AF324495
	5-TCCTGATTTCATCTTGCCACC-3′	
Cyclophilin	5′-CAAATGCTGGACCAAACACA-3′	X17105
	5′-CAGTCTTGGCGGTGCAGAT-3′	

### Western blot

Approximately 150 mg of liver tissue was homogenized adequately prepared for Western blotting as described previously [21]. An aliquot (50 µg) of sample proteins were denatured in sample buffer containing SDS and β-mercaptoethanol, separated on a 4–20% gradient SDS-PAGE gel, and electroblotted onto nitrocellulose membranes. Nonspecific binding sites of the membranes were blocked using defatted milk proteins followed by the addition of antibodies against LDLR (Santa Cruz, CA, USA). The relative amount of primary antibody was detected with peroxidase-conjugated secondary antibody. Densitometry was quantified using the BioRad Quantity one software. Similar procedures were carried out with antibodies against cholesterol-7α- hydroxylase (cyp7α-1) (Santa Cruz, CA, USA), ABCG5 (Santa Cruz, CA, USA), ABCG8 (Santa Cruz, CA, USA), NPC1L1 (Santa Cruz, CA, USA) and β-actin (Santa Cruz, CA, USA). The protein expression was expressed as a ratio of the target protein to β-actin.

### Histological examination

Liver tissues were rinsed with saline water (0.9%, w/w) and fixed in methyl aldehyde solution (1∶9, 0.01 M, pH 7.4, NaH2PO3/Na2HPO 3), embedded in paraffin wax. Sections of 5 µm thickness were affixed to slides, deparaffinized, dehydrated and then stained with haematoxylin and eosin using routine methods. Stained sections were observed under an optical microscope and photographs were taken to record the histological change.

### Statistical analysis

Results were expressed as mean±standard deviation. One-way ANOVA analysis of variance was used to analyze plasma and liver lipids, plasma concentration of liver enzyme, cholesterol absorption rates, cholesterol synthesis rates, and gene and protein expressions using SPSS 17.0 (SPSS Inc., Chicago, IL, USA). Relationships between plasma lipid concentrations and intestinal cholesterol absorption rates were analyzed using Pearson’s correlation coefficients. Significance level was set up at p<0.05.

## Results

All 24 hamsters survived the 6-month experiment. The mean initial and final body weights for all 24 hamsters were 205±5 g and 252±7 g,respectively. And no significant differences were observed between groups.

### Effect of APS on plasma lipoproteins

**Figure 1:**
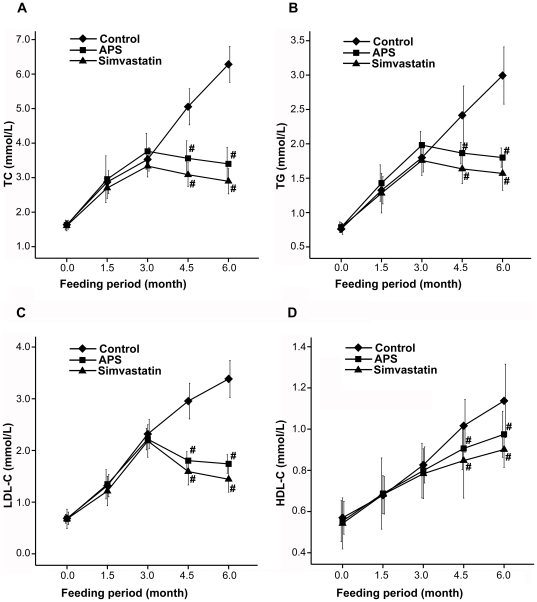
Changes of plasma lipids in response to APS and simvastatin treatments. APS and simvastatin significantly lowered total cholesterol (TC), triglycerides (TG), high-density lipoprotein –cholesterol (HDL-C) and low-density lipoprotein-cholesterol (LDL-C) concentrations compared to the control. # P<0.05 compared with control group.

Before APS treatment, animals were fed a high fat diet for three months, which significantly increased plasma TC, TG, LDL-C and HDL-C and no intergroup differences were found (P>0.05). Treatments of these hyperlipidemic animals with APS and simvastatin for three months resulted in marked decreases in plasma TC, TG, and LDL-C concentrations. Compared to the control, APS and simvastatin treatments significantly (P<0.05) decreased plasma TC by 45.8% and 53.8%, TG by 30% and34.2%, LDL-C by 47.4% and 56.5%, respectively. Nevertheless, the difference failed to achieve statistical significance between APS and Simvastatin group. Both APS and simvastatin reduced (p<0.05) plasma HDL-C levels compared to the control group (*Figure 1*).

### APS increased fecal bile acid and neutral sterol excretion

To determine the effect of APS on whole-body cholesterol turnover, the daily output of bile acids and neutral sterols in the feces was measured. There were no significant differences between groups for any fecal steroids prior to treatment. However, after three months, the fecal bile acid and neutral sterol excretion were significantly (P<0.05) higher in the APS groups than in the Simvastatin and control group (*Table 2*). No significant differences were found between Simvastatin and control group.

**Table 2:** Effect of APS and simvastatin on fecal excrection of bile acids and neutral sterol.

Fecal lipid	Feeding period	Control (n = 8)	APS (n = 8)	Simvastatin (n = 8)
bile acids	Month 0	12.24±2.89	13.35±3.17	12.46±2.29
(µmol/d/100 g BW)	Month 3	23.98±4.11	24.87±4.76	22.01±4.19
	Month 6	37.85±7.14	51.99±8.17^#^ *	29.43±5.16
neutral sterols	Month 0	18.78±2.95	20.14±3.43	19.51±3.19
(µmol/d/100 g BW)	Month 3	48.67±7.70	49.07±6.76	47.02±6.36
	Month 6	75.07±8.41	92.23±8.10^#^ *	61.70±7.03

The daily total bile acids and neutral sterols were measured and expressed per 100 g body weight (BW). Values are means±SDs of 8 animals per group.

# P<0.05 compared with Control group,

*P<0.05 compared with Simvastatin group.

### APS inhibited intestinal fractional cholesterol absorption

**Figure 2:**
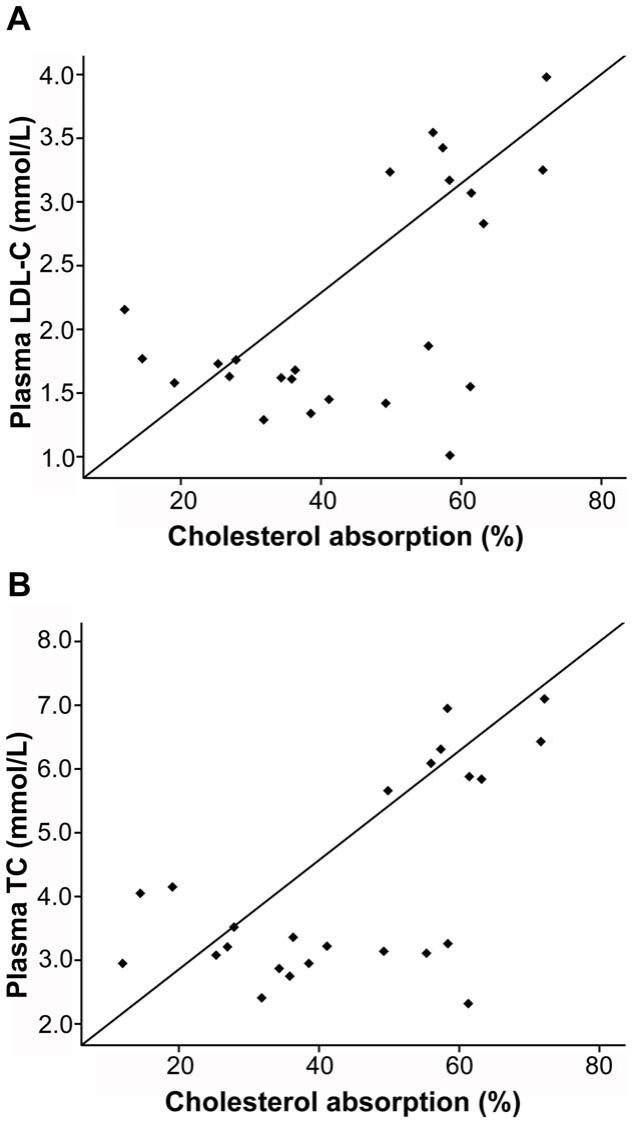
Relationships between plasma lipids and intestinal cholesterol absorption. Intestinal cholesterol absorption rates are significantly correlated with the plasma LDL-C (A) and TC (B) levels, respectively.

To understand the mechanism by which the treatments affect plasma cholesterol levels, we have measured fractional cholesterol absorption rate in all animals of each group using the dual plasma stable isotope ratio method. Compared with the control, APS inhibited (p<0.05) fractional cholesterol absorption by 59.9%. However, there is no difference between Simvastatin and Control group *(* *Table 3* *)*. Fractional cholesterol absorption rates were strongly associated with plasma TC (r = 0.723, p<0.0001; *Figure 2A*) and LDL-C (r = 0.783, p<0.0001; *Figure 2B*).

**Table 3:** Effect of APS on fractional cholesterol absorption and cholesterol synthesis rates in hamsters.

Group	Absorption (%)	Synthesis (nmol/h/g tissue)
Control	61.23±7.67	278±45
APS	24.53±8.78^#^ *	371±53^#^ *
Simvastatin	56.44±11.12	220±39

Values are means±SD of 8 animals per group.

# P<0.05 compared with Control group,

*P<0.05 compared with Simvastatin group.

### APS up-regulated cholesterol synthesis rates

As reported previously [22], cholesterol synthesis changed reciprocally with the internal cholesterol absorption. To examine the effect of APS on cholesterol synthesis in the liver, we measured in vivo cholesterol synthesis rate in all animals using tritiated water. Expectedly, compared to Simvastatin and Control group, the rate of hepatic cholesterol synthesis in APS group was increased by 33.4% (p<0.05) and 66.8% (p<0.05), respectively (*Table 3*).

### Effect of APS on cholesterol metabolism associated gene and protein expressions in the liver and small intestine

**Figure 3:**
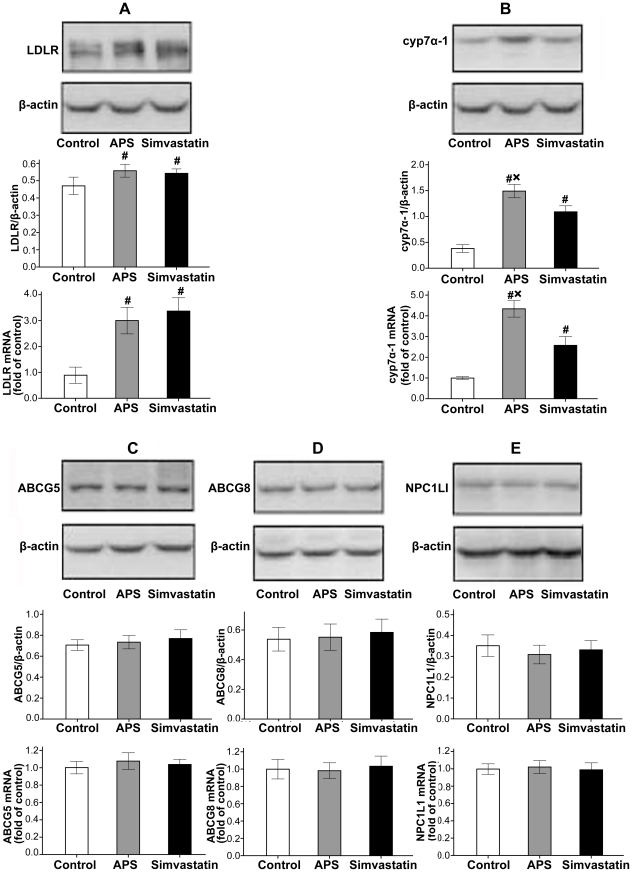
Cholesterol metabolism associated gene and protein expressions. Representative Western blot and group data depicting LDLR protein and mRNA abundance (A), cyp7α-1 protein and mRNA abundance (B) in livers, ABCG5 protein and mRNA abundance(C), ABCG8 protein and mRNA abundance(D), NPC1L1 protein and mRNA abundance(E) in intestines of hamsters. N = 8 per group. # P<0.05 compared with Control group, *P<0.05 compared with Simvastatin group.

In addition to cholesterol absorption and synthesis, quantitative real-time PCR and western blot were used to measure the expression of key genes of cholesterol metabolism in the livers (*Figure 3*). LDLR is involved in liver clearance of LDL-C from circulation. The present study showed that mRNA and protein abundance of LDLR was higher in APS and simvastatin treated hamsters than the control. The difference did not reach statistical significance between APS group and Simvastatin group. The current study shows both APS and simvastatin elevated hepatic cyp7α-1 gene expression significantly compared to the control (P<0.001), whereas APS demonstrated superior ability in this effect (P<0.05).

In order to understand how APS affect cholesterol absorption, we measured their gene and protein expressions in the small intestine. We found that APS did not show a significant effect on the mRNA and protein levels of ABCG5, ABCG8 or NPC1L1 in the small intestine. Similarly, simvastatin did not affect cholesterol transport associated gene and protein expressions compared to the control (*Figure 3*).

### Effect of APS on liver HMG-CoA reductase activity

After 3-month treatment, HMG-CoA reductase activity in APS group, Simvastatin group and control group were 36.24±5.62 pmole/min/mg protein, 12.60±2.03 pmole/min/mg protein and 22.86±6.45 pmole/min/mg protein, respectively. Statistical analysis found significant difference among the three groups. Relative to the control and Simvastatin group, APS significantly increased the HMG-CoA reductase activity by 58.5% (P<0.05) and 1.87 fold (P<0.05), respectively.

### Effect of APS on liver lipids, enzymes and histopathology

**Figure 4:**
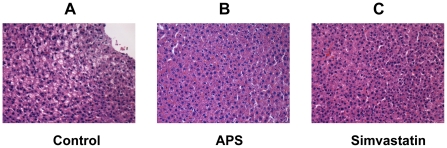
HE staining of liver tissue from different groups (Original magnification, ×200). A: control group; B: APS group; C: Simvastatin group. Livers of APS and Simvastatin group show mild fatty degeneration. In contrast, livers of control rats showed severe fatty degeneration, extensive hepatocytes hypertrophy, oedema, and vacuolization of the hepatocyte cytoplasm.

In comparison to the control, APS and simvastatin significantly (P<0.001) reduced liver TC by 45.6% and 41.9%, and TG by 41.8% and 40.9%, respectively (*Table 4* *)*. There is no significant difference found between APS and Simvastatin group. To determine whether APS have hepatotoxic effect on hamsters, we have measured the plasma concentration of ALT and AST and performed histological examination of the livers. Our study shows APS and simvastatin significantly decreased the concentrations of plasma ALT and AST (P<0.05), and markedly ameliorated liver fatty degeneration compared to the control (*Figure 4*).

**Table 4:** Effect of APS liver lipids and plasma concentration of ALT in hamsters.

Group	Liver total cholesterol	Liver triglycerides	Plasma ALT	Plasma AST
	(mg/g tissue)	(mg/g tissue)	(U/L)	(U/L)
Control	20.09±3.23	13.12±1.97	137.25±19.82	168.35±23.56
APS	10.97±1.94#	7.64±0.83#	56.86±8.86#	76.47±12.91#
Simvastatin	11.67±2.07#	7.75±0.95#	59.63±9.58#	80.48±14.17#

Values are means±SDs of 8 animals per group.

# P<0.05 compared with Control group.

## Discussion

Inhibiting HMG-CoA reductase activity and increasing hepatic LDLR expression are primary mechanisms of statins therapy for hyperlipidemia. Here, we identify a new cholesterol-lowering drug, APS, that effectively reduces plasma cholesterol to levels comparable to that of statins, but may work through different mechanisms. In our study, a 45.8% reduction of TC, 30% reduction of TG, and 47.4% reduction of LDL-C were achieved in hyperlipidemic hamsters after 3-month treatment of APS, similar to statins that lowered LDL-C by 56.5%. Previous study by Wu CZ et al. showed Ogi-Keishi-Gomotsu-To-Ka-Kojin (OKGK), a Chinese medicine composite that contains a small amount of APS (10.07%) may also exert a hypolipidemic effect in hamsters [23]. Yet they did not make clear which ingredient really works, as Panax ginseng, a crude drug of OKGK was also reported to show antihypercholesterolemic action in human, chicken and rat [24], [25].

The small intestines are implicated in regulating cholesterol homeostasis through affecting cholesterol absorption. An inhibition of intestinal absorption results in lower levels of circulating cholesterol. As ezetimibe and sitosterol, which act directly at the level of intestine, lower plasma cholesterol by inhibiting intestinal fractional cholesterol absorption [26], [27]. Our results revealed that APS led to a profound reduction in intestinal cholesterol absorption and a markedly accelerated rate of fecal neutral sterol excretion. Correlation between cholesterol absorption rates and plasma TC or LDL-C concentrations were also observed, suggesting a hitherto unknown mechanism that APS reduces plasma cholesterol by interfering with the intestinal cholesterol absorption.

Previous researchers have identified Niemann-Pick C1 like 1 (NPC1L1) as a cholesterol uptake transporter [28] and the ATP-binding cassette (ABC) proteins ABCG5 and ABCG8 as cholesterol efflux transporter [29]. However, our studies indicated that the sterol transporters, ABCG5, ABCG8, and NPC1L1 in the small intestine were not involved in the inhibition of cholesterol absorption. It has been proved that the viscosity associated with dietary soluble polysaccharides interferes with cholesterol absorption by directly binding cholesterol within the intestine [10], interfering with the diffusion of cholesterol toward the epithelial cell surface [11], or reducing the capacity of micelles to incorporate cholesterol [12]. We thus propose the reduction of cholesterol absorption in response to APS maybe attributed to its viscosity, with little regulation of genes associated with cholesterol transport.

Cholesterol is mainly eliminated from the body via conversion to bile acids, and the rate-limiting enzyme of the process is cyp7α-1 [30]. It has been reported that dietary soluble polysaccharides decreased serum cholesterol by altering the composition of the enterohepatic bile acid pool and increasing the fecal loss of total bile acids [13], [14]. In response to increased fecal bile acid excretion, there occurs a compensatory increase in bile acid synthesis [31]. In this study, we reported for the first time that APS produced a marked increase in excretion of fecal bile acids, in accordance with our observations that the hepatic cyp7α-1 mRNA expression was significantly elevated in response to APS. A further test with Western blotting also revealed that the APS stimulated cyp7α-1 protein expression. The effect on bile acid metabolism would support the conclusion that APS lowers blood cholesterol partly by increasing bile acid excretion. However, additional studies are required to further investigate the mechanism by which APS inhibits cholesterol absorption and increases bile acid excretion.

Increased cholesterol synthesis by cholesterol absorption inhibitors has been reported in many previous studies [22], [27]. We observed for the first time that APS also increased cholesterol synthesis, different from that of statins. Cholesterol synthesis increases reciprocally to the reduction of absorption as a compensatory metabolic response [20], [32], as indicated by an increase in the fecal excretion of neutral sterols and liver HMG-CoA reductase activity in our study. It can be speculated that the increase in cholesterol synthesis is mainly due to de novo hepatic cholesterol. As 45.8% of plasma TC, 30% of plasma TG, and 47.4% of plasma LDL-C, and 45.6% of liver TC, 41.8% of liver TG was reduced in hamsters treated with 0.25 g/kg/d of APS, it is also possible that plasma cholesterol was decreased to minimum that hamster have to maintain normal physiological function by up-regulating cholesterol synthesis. Although the reciprocal increase of cholesterol synthesis might have compromised partly the cholesterol lowering efficacy of the treatments, our data and many previous reports demonstrated plasma cholesterol was still significantly reduced [21], [22], [32], [33]. The results suggest that supplementation of APS for 3 months may be beneficial to liver health and function, which is supported by a significant decrease of ALT and AST and amelioration of liver fatty degeneration in hamsters under the experimental conditions.

The expression of liver LDLR regulates plasma LDL-C homeostasis in both human and hamsters. Increased hepatic LDLR expression results in improved clearance of plasma LDL-C through receptor-mediated endocytosis [34], [35]. The activation of LDLR gene expression through depletion of intracellular cholesterol is the principal working mechanism of statins [36]. In this study, we demonstrated that APS had strong activities in stimulating hepatic LDLR mRNA expression. The effect of APS on LDLR was further confirmed with Western blotting. Again, APS significantly increased LDLR protein on hepatic cell surface. The specific mechanism for this regulation is unknown, but it could be possible that, similar to simvastatin, APS works through a negative feedback mechanism by depleting intracellular cholesterol pools. Our results revealed the decrease of plasma LDL-C was in agreement with the up-regulation of LDLR, which suggests APS may regulate cholesterol homeostasis partially through inducing LDL-R expression.

It is of note that a significant reduction of HDL-C was seen in the current study after APS or simvastatin treatment. Other studies point out HDL is in high proportion of plasma cholesterol in hamsters. Consequently, a reduction of TC is usually accompanied by a decrease of HDL-C [26], [37], [38]. On the other hand, HDL-C of hamsters contains high concentration of apo E and may thus be cleared by LDL-R [39]. However, this effect is generally not translated to humans [22].

In conclusion, these findings strongly suggest APS is a promising novel natural health hypolipidemic drug that may act by multiple mechanisms. APS lowers plasma cholesterol through a combination of inhibiting fractional cholesterol absorption, increasing fecal bile acid excretion, up-regulating hepatic LDL-R, cyp7α-1 gene expression. This study indicates that if proven in human trials, APS would provide an alternative to statins for patients with hyperlipidemia, atherosclerosis or coronary heart disease.
